# Supplementary material for: Multi‐dataset identification of innovative feature genes and molecular mechanisms in keratoconus
Source: J Cell Mol Med. 2024 Sep 19;28(18):e70079. doi: 10.1111/jcmm.70079 (PMC11412914; doi:10.1111/jcmm.70079)
Supplement: Supplementary file 2 — Material S2. [file JCMM-28-e70079-s002.zip › 7.GSEA analysis/1.ARL11/KEGG_HEMATOPOIETIC_CELL_LINEAGE.html]

Details for gene set KEGG\_HEMATOPOIETIC\_CELL\_LINEAGE[GSEA]

|  || Dataset | ARL11.group.cls#h\_versus\_l |
| Phenotype | group.cls#h\_versus\_l |
| Upregulated in class | h |
| GeneSet | KEGG\_HEMATOPOIETIC\_CELL\_LINEAGE |
| Enrichment Score (ES) | 0.28420007 |
| Normalized Enrichment Score (NES) | 0.86428183 |
| Nominal p-value | 0.67695475 |
| FDR q-value | 1.0 |
| FWER p-Value | 1.0 |
Table: GSEA Results Summary

  

Fig 1: Enrichment plot: KEGG\_HEMATOPOIETIC\_CELL\_LINEAGE      
 Profile of the Running ES Score & Positions of GeneSet Members on the Rank Ordered List

  

| SYMBOL | TITLE | RANK IN GENE LIST | RANK METRIC SCORE | RUNNING ES | CORE ENRICHMENT || 1 | IL7R | na | 136 | 0.619 | 0.0421 | Yes |
| 2 | ITGA4 | na | 272 | 0.550 | 0.0792 | Yes |
| 3 | FCER2 | na | 343 | 0.521 | 0.1160 | Yes |
| 4 | CD8B | na | 580 | 0.460 | 0.1434 | Yes |
| 5 | CD3G | na | 1113 | 0.375 | 0.1559 | Yes |
| 6 | IL3RA | na | 1221 | 0.364 | 0.1800 | Yes |
| 7 | CR1 | na | 2122 | 0.287 | 0.1754 | Yes |
| 8 | CSF1R | na | 2453 | 0.268 | 0.1858 | Yes |
| 9 | HLA-DRB5 | na | 2778 | 0.251 | 0.1952 | Yes |
| 10 | CD19 | na | 3009 | 0.240 | 0.2064 | Yes |
| 11 | IL4R | na | 3015 | 0.240 | 0.2241 | Yes |
| 12 | FCGR1A | na | 3110 | 0.236 | 0.2389 | Yes |
| 13 | CD2 | na | 3166 | 0.233 | 0.2547 | Yes |
| 14 | IL3 | na | 3483 | 0.220 | 0.2620 | Yes |
| 15 | CD22 | na | 3567 | 0.217 | 0.2757 | Yes |
| 16 | IL7 | na | 3809 | 0.208 | 0.2842 | Yes |
| 17 | CD3E | na | 4816 | 0.171 | 0.2679 | No |
| 18 | CD1A | na | 6054 | 0.136 | 0.2423 | No |
| 19 | CD5 | na | 6155 | 0.133 | 0.2493 | No |
| 20 | IL2RA | na | 6518 | 0.124 | 0.2481 | No |
| 21 | IL6 | na | 6613 | 0.121 | 0.2544 | No |
| 22 | CD3D | na | 6811 | 0.116 | 0.2574 | No |
| 23 | HLA-DRB4 | na | 7218 | 0.107 | 0.2537 | No |
| 24 | FLT3 | na | 7449 | 0.103 | 0.2547 | No |
| 25 | CD14 | na | 7503 | 0.102 | 0.2607 | No |
| 26 | EPO | na | 7618 | 0.099 | 0.2648 | No |
| 27 | HLA-DRA | na | 7656 | 0.099 | 0.2711 | No |
| 28 | FLT3LG | na | 7704 | 0.098 | 0.2770 | No |
| 29 | HLA-DRB1 | na | 7965 | 0.093 | 0.2764 | No |
| 30 | CD8A | na | 8817 | 0.078 | 0.2577 | No |
| 31 | CD4 | na | 11083 | 0.047 | 0.1957 | No |
| 32 | KITLG | na | 11152 | 0.046 | 0.1972 | No |
| 33 | GP1BB | na | 11425 | 0.042 | 0.1925 | No |
| 34 | TFRC | na | 11569 | 0.041 | 0.1914 | No |
| 35 | CD1E | na | 13132 | 0.026 | 0.1482 | No |
| 36 | EPOR | na | 13761 | 0.021 | 0.1317 | No |
| 37 | CD9 | na | 14899 | 0.012 | 0.0997 | No |
| 38 | CSF1 | na | 15339 | 0.009 | 0.0877 | No |
| 39 | ITGAM | na | 15359 | 0.009 | 0.0878 | No |
| 40 | IL11RA | na | 15527 | 0.007 | 0.0835 | No |
| 41 | KIT | na | 16102 | 0.003 | 0.0672 | No |
| 42 | IL5RA | na | 16947 | -0.003 | 0.0430 | No |
| 43 | GYPA | na | 18841 | -0.016 | -0.0105 | No |
| 44 | ITGA2 | na | 19176 | -0.018 | -0.0187 | No |
| 45 | ITGA3 | na | 19278 | -0.019 | -0.0203 | No |
| 46 | GP9 | na | 19306 | -0.019 | -0.0196 | No |
| 47 | HLA-DRB3 | na | 19647 | -0.021 | -0.0278 | No |
| 48 | ITGA2B | na | 19828 | -0.023 | -0.0313 | No |
| 49 | IL6R | na | 20441 | -0.027 | -0.0470 | No |
| 50 | DNTT | na | 20864 | -0.030 | -0.0569 | No |
| 51 | CD33 | na | 21252 | -0.033 | -0.0656 | No |
| 52 | ITGA6 | na | 22097 | -0.041 | -0.0869 | No |
| 53 | CR2 | na | 22325 | -0.043 | -0.0903 | No |
| 54 | TNF | na | 22702 | -0.047 | -0.0976 | No |
| 55 | CD59 | na | 22911 | -0.049 | -0.1000 | No |
| 56 | TPO | na | 23798 | -0.060 | -0.1211 | No |
| 57 | CD7 | na | 24617 | -0.071 | -0.1394 | No |
| 58 | CD55 | na | 24695 | -0.072 | -0.1363 | No |
| 59 | CSF3R | na | 24746 | -0.073 | -0.1323 | No |
| 60 | CD1B | na | 24853 | -0.074 | -0.1299 | No |
| 61 | IL9R | na | 25644 | -0.085 | -0.1464 | No |
| 62 | ANPEP | na | 26145 | -0.093 | -0.1539 | No |
| 63 | CD44 | na | 26158 | -0.093 | -0.1473 | No |
| 64 | CD36 | na | 26649 | -0.102 | -0.1539 | No |
| 65 | CD38 | na | 26661 | -0.102 | -0.1467 | No |
| 66 | GP1BA | na | 27425 | -0.117 | -0.1600 | No |
| 67 | CD34 | na | 28207 | -0.137 | -0.1723 | No |
| 68 | IL1B | na | 28530 | -0.144 | -0.1709 | No |
| 69 | IL1A | na | 28564 | -0.145 | -0.1610 | No |
| 70 | ITGB3 | na | 29449 | -0.171 | -0.1738 | No |
| 71 | MME | na | 29457 | -0.171 | -0.1613 | No |
| 72 | CD1D | na | 29529 | -0.173 | -0.1505 | No |
| 73 | CSF3 | na | 29731 | -0.180 | -0.1429 | No |
| 74 | ITGA1 | na | 29976 | -0.187 | -0.1361 | No |
| 75 | CD37 | na | 30019 | -0.188 | -0.1233 | No |
| 76 | CSF2 | na | 30130 | -0.191 | -0.1122 | No |
| 77 | IL1R1 | na | 30266 | -0.196 | -0.1014 | No |
| 78 | IL1R2 | na | 30605 | -0.208 | -0.0957 | No |
| 79 | CD1C | na | 31165 | -0.227 | -0.0950 | No |
| 80 | GP5 | na | 32019 | -0.261 | -0.1002 | No |
| 81 | THPO | na | 32264 | -0.273 | -0.0869 | No |
| 82 | ITGA5 | na | 32559 | -0.291 | -0.0737 | No |
| 83 | IL5 | na | 32563 | -0.292 | -0.0521 | No |
| 84 | CSF2RA | na | 32761 | -0.305 | -0.0350 | No |
| 85 | IL4 | na | 33709 | -0.380 | -0.0341 | No |
| 86 | MS4A1 | na | 33710 | -0.380 | -0.0057 | No |
| 87 | IL11 | na | 34287 | -0.471 | 0.0127 | No |
Table: GSEA details [plain text format]

  

Fig 2: KEGG\_HEMATOPOIETIC\_CELL\_LINEAGE      
 Blue-Pink O' Gram in the Space of the Analyzed GeneSet

  

Fig 3: KEGG\_HEMATOPOIETIC\_CELL\_LINEAGE: Random ES distribution      
 Gene set null distribution of ES for **KEGG\_HEMATOPOIETIC\_CELL\_LINEAGE**
